# Can a multi-level intervention approach, combining behavioural disciplines, novel technology and incentives increase physical activity at population-level?

**DOI:** 10.1186/s12889-020-10092-x

**Published:** 2021-01-11

**Authors:** Ling Chew, Isabel Tavitian-Exley, Nicole Lim, Alice Ong

**Affiliations:** Singapore Health Promotion Board, Singapore, Singapore

**Keywords:** Physical activity, Modifiable risk factor, Non-communicable diseases, Wearable technology, Implementation science, Natural experiment, Southeast Asia

## Abstract

**Background:**

Despite a global call for action and growing burden of non-communicable diseases (NCD) associated with physical inactivity, effective interventions to increase community-wide physical activity (PA) remain few. NCDs accounted for 80% of Singapore’s disease burden (2015) and yet 40% of Singaporeans did not meet minimum recommended weekly PA despite evidence of the benefits to cardiorespiratory health, diabetes and cancer prevention*.*

**Methods:**

A large-scale public health intervention was initiated in 2015 to increase population-level PA through incidental daily walking. Intervention components included fitness trackers, redeemable rewards and gamification, implemented in a mutually-reinforcing manner within an eco-system supportive of PA and informed by real-time data analytics. Mean daily step count at baseline and post-intervention were compared across periods, and the influence of participant sub-groups characteristics on overall results, using significance tests. Standards for Reporting on Implementation Studies (StaRI) were adhered to.

**Results:**

Intervention reach increased fourfold from 129,677 participants in wave 1 (2015–16) to 690,233 in wave 3 (2017–18) amounting to a total of 1,184,410 Step Challenge participations. Mean days of fitness tracker use increased from 2.4 to 5.0 days/week among participants completing the Challenge in wave 1 and from 5.3 to 6.0 days/week in wave 3. The mean number of daily steps between pre-Challenge and Challenge periods increased by 4163 (sd=1360; *p*< 0.001) in wave 1, by 2242 (sd=334; p< 0.001) in wave 2 and by 1645 steps/day (sd=54; p< 0.001) in wave 3. Mean daily step increases between wave 1 and 3 also suggest that incidental PA was maintained, a finding supported by a 2017 national population survey showing that incidental PA among adults increased from 5% in 2010 to 14% in 2017 while moderate-intensity PA increased from 5 to 10% over the same period.

**Conclusion:**

Population-level PA was effectively increased through multi-level interventions integrating technology, behavioural economics, gamification, marketing, communications and community linkages within a supportive context- and climate-appropriate environment. Responsive data analytics were instrumental to strengthen implementation by tailoring modalities that maximise effectiveness at population-level. Further analyses are needed to explore potential barriers, challenges or unmet needs in sub-groups with lower uptake to tailor future interventions for greater reach and impact.

**Supplementary Information:**

The online version contains supplementary material available at 10.1186/s12889-020-10092-x.

## Contributions to the literature

- Despite a global call for action and growing burden of non-communicable diseases (NCD) associated with physical inactivity, effective interventions on a sustained basis to increase community-wide physical activity (PA) remain few.
- Using an implementation science approach, we enrolled almost a million adults of all ages in a multi-level public health experiment rewarding manageable increments in daily walking over several months, within an eco-system supportive of PA and informed by on-going analytics.
- Daily PA was increased at population-level by integrating technology, behavioural economics, gamification, marketing and communications interventions with community linkages in a context- (and climate-) appropriate environment supportive of PA.
- Limitations are acknowledged.

## Background

The increase in non-communicable diseases worldwide has become an urgent and multi-faceted challenge, which increases in physical activity (PA) can help reverse [1–3]*.* Sedentary lifestyles are a major risk factor for cardiovascular disease, cancer and obesity and physical inactivity is estimated to account for up to 30% of deaths from ischaemic heart disease and 6–10% of all deaths from non-communicable diseases (NCD) worldwide [4, 5]*.* Morbidity related to PA is associated with heavy health and economic costs and places a burden on families, societies and health systems [4, 6, 7]*.*

The rapid epidemiological transition from communicable to non-communicable diseases in countries experiencing fast economic growth and social change has led to equally rapid increases and shifts in health care and prevention needs [7–9]*.* In 2015, NCDs accounted for 80.3% of the disease burden in Singapore, almost a third of which were cardiovascular diseases and cancers (27.5%) [6]*.* Despite several years of health promotion, 39% of Singaporean adults aged 18–69 years and nine in ten secondary school students did not meet the minimum recommended weekly PA,^1^ citing lack of motivation or time to exercise [10]*.* The gap between knowledge and practice and, the rising burden of NCDs and their associated costs, catalysed a shift to re-think new approaches to promote healthy behaviours at population-level [10, 11]*.*

Despite robust evidence that PA is a direct contributor to cardiorespiratory health and to preventing diabetes and certain cancers, increasing PA at population-level has been challenging [12–14]. Recognising that changing unhealthy behaviours requires more than personal motivation, interventions combining behavioural, informational, environmental and policy components have been implemented with varying degrees of success [12, 14–17]*.* Financial incentives, fitness trackers and interventions that introduce a gamification component have shown to increase PA in adults [18–22]*.* A systematic review found that pedometers improved PA by 26% on average while another trial found that wrist-worn fitness trackers compared to cash incentives only were associated with maintaining step increases at 12-months [19, 23]*.* Evidence that trackers may contribute to increases in PA among adults and developments in smartphone technology has opened up opportunities relevant to a well-connected, urban setting with high smartphone penetration such as Singapore [21, 24, 25].

The driving force behind the promotion of health and healthy living in Singapore is the Health Promotion Board (HPB), a quasi-government agency set up in 2001 with dedicated funding to drive health promotion programmes [26]*.* In 2014, the HPB adopted a systemic approach to health promotion in which multi-component interventions are combined purposefully to create an environment that enables and supports healthy habits by offering healthy living options as a default [11, 26].

In 2015, HPB initiated a multi-level public health intervention to promote PA. HPB hypothesised that PA could be increased at population-level by promoting small and manageable increments in daily walking at individual-level, facilitated through several mutually-reinforcing behavioural, economic, technology, environmental and gamification-related intervention components. This first large-scale population-based intervention aimed to encourage people to increase regular PA and daily walking, while testing implementation modalities such as rewards and incentive structures, social marketing and advertising campaigns to mobilise and keep participants engaged. Daily step increase was the primary outcome measure of PA.

## Methods

### Study population

The eligibility criteria to participate in this intervention were residing in Singapore, being 17 years or older, able to provide consent to readiness for PA and to the use of aggregated data for analysis by HPB. Three separate recruitment periods were conducted between November 2015 and May 2016 (Wave 1), October 2016 and February 2017 (Wave 2) and October 2017 and Feb 2018 (Wave 3). Wave participants were followed up for up to 40 weeks. There was no restriction to participation in a subsequent wave.

### Conceptual approach and design

The overall design, data collection, planning and execution adopted an implementation science approach using real-time data analytics [27–29]*.* Several promising behaviour-change intervention components including wearable fitness trackers, incentives and gaming principles formed the core intervention of the National Steps Challenge (the Challenge). The implementation strategy was grounded in a socio-ecological framework approach that built on HPB’s ground presence and supportive social, economic and structural environment to enable individual and population-level PA [30–34].

### Interventions and modalities

Eligible participants were given a commercial grade wrist-worn fitness tracker with built-in accelerometer then paired to a dedicated smartphone application “Healthy365” (H365). This provided a user-friendly interface to record daily steps, give users feedback on their step count and activate neural reward feedback mechanisms to reinforce positive behaviours [35, 36]*.* The intervention design was guided by the EAST framework to make it **E**asy, **A**ttractive, **S**ocial and **T**imely and maximise its effectiveness [37, 38]. Participants who achieved modest daily step increases over set periods of time were rewarded for *easy* targets of 5000, 7500 and 10,000 daily steps, based on evidence that even small increases in physical activity can improve health [39–41]. The free wearable watch-tracker and redeemable voucher received by participants after signing up increased desirability, became associated with the National Step Challenge *(****S****ocial)* and helped increased awareness and interest for it (***T****imely*)*.* Smartphone technology made step tracking **E**asy and increased the timeliness and immediacy of the feedback, nudges and rewards, thus increasing chances of sustaining behaviour change [36].

#### Behavioural science, economics and gamification

The fitness trackers provided real-time data to individuals on their step count and to HPB on the participating cohort. To introduce an element of fun and to sustain interest among participants over time, a tiered system offered redeemable points over 5 months during which habituation is estimated to occur (Table 1) [35]. The rewards structure drew on evidence from behavioural economics that financial incentives and phone-based game applications contributed to increasing PA among adults and 18–35 years olds for a period of up to 5 weeks [16, 18–20, 42]*.*

**Table 1:** Reward structure and characteristics

	NSC wave 1	NSC wave 2	NSC wave 3
**Reward structure**	3 tiers of guaranteed rewards	6 tiers of guaranteed rewards	6 tiers of guaranteed rewards
**Rewards type** Points to be redeemed for transport, food or retail purchases.	Redeemable points for achieving 5000, 7500 and 10,000 steps/day over period equivalent to 60 days > 10,000 steps - Capped at 60 points/day.	Redeemable points for achieving 5000, 7500, 10,000 steps/day over a period, equivalent to > 116 days of > 10,000 steps - Capped at 60 points/day (broken up into more tiers with smaller reward for double the effort).	Redeemable points for achieving 5000, 7500, 10,000 steps/day over a period, equivalent to > 120 days of > 10,000 steps (as in NSC2) - points capped at 40 points/day (harder to achieve each milestone).
	▪ Lucky draw ▪ Allow goal setting for participants ▪ Create positive reinforcement at each milestone	▪ Modest lucky draw prizes and more expensive top prize offered (i.e. air ticket to New Zealand). ▪ Monthly **thematic challenge** in the form of fun activities such as walking trails and treasure hunts around shopping malls to keep participants engaged throughout duration of the NSC *(new)*	▪ Smaller lucky draw prizes and more expensive top prize offered (i.e. SIA Business Class tickets to Stockholm). ▪ More thematic challenges; ▪ **Target setting** element introduced to encourage commitment (**pledge & win**): Pledge to achieve a daily target number of steps over a stipulated number of days to win special prizes.
**Fitness tracker used** (commercial grade accelerometer)	Actxa™ Stride wrist-worn step trackers	Actxa™ Stride fitness trackers (steps + heart rate), AB fitness, Omniband	Actxa™ Stride fitness trackers (steps + heart rate + PA intensity), Careach, Mova, Skytech, Xstep, Tempo.

*Source:* Health Promotion Board, 2019. *NSC* National Step Challenge, *PA* Physical Activity

Guaranteed rewards were given for daily step targets of 5000, 7500 and 10,000 over set periods of time. These encouraged participants to increase their daily steps and to maintain increases for the duration of the Challenge [43]. Participants could also enter a lucky draw for which the odds of winning were linked to the number of steps achieved*.* In addition to attractive lucky draw and thematic challenges prizes, sponsors also offered branded lifestyle and sportswear.

Several modalities were introduced during implementation to address declines in engagement or interest observed in the data. Thematic challenges consisting of fun activities (ie. walking trails or treasure hunts around shopping malls) were introduced in wave 2 to maintain participants engagement throughout, while targeted challenges were introduced to better engage youth, workplaces and/or local communities. In wave 3, target-setting was introduced for those completing all step tiers. This took the form of a pledge to achieve a daily step target over a stipulated number of days, to win special prizes.

Social marketing was used to generate population-level interest prior to implementation and mobilise participants through promotional activities in public spaces, including live entertainment events in popular transit areas and shopping malls where people could register for the Challenge, obtain and “activate” the fitness trackers to synchronise with H365 [43]. Venues selected for these events were high pedestrian traffic areas, including transit areas, public spaces and shopping malls. Participants tracked their progress on the H365 application which also provided positive reinforcement messages for each milestone completed. Monthly thematic challenges were introduced in wave 2, to sustain or re-ignite efforts after the novelty had worn off or at times when interest appeared to weaken. These were complemented by a nation-wide media advertising campaign and publicity in workplaces, educational and community settings.

Wider reach and participation were enabled through partnerships with the commercial sector such as pharmacies and mall operators, and facilitated by improvements in the physical environment such as sheltered walkways and park connectors [44]. Mall operators offered venues and marketing support for recruitment events while participants without smartphones^2^ could synchronise their step count in pharmacy-based Healthy365 kiosks throughout the island [24].

Throughout this period, improvements in urban walkability were simultaneously carried out by infrastructure authorities to facilitate walking and PA. Between 2014 and 2018, under the Land Transport Authority’s “last mile initiative”, the network of covered walkways protecting pedestrians from the sun and rain between bus stops and housing complexes or offices expanded by 200 km [44]. Walking and cycling tracks in the National Parks Authority island-wide connector network also increased to 313 km [45]*.*

#### Data collection, analytics and feedback

The intervention was designed to generate on-going population data analytics as well as individual feedback in order to continuously inform implementation, allow timely adjustments during the Challenge and to enable pre- and post-intervention evaluation. Individual and population-level data on walking steps, patterns and behaviours were generated, and adjustments made when required in the following wave - for example if targets were too hard or easy to reach or if the team detected unusual usage. The commercial-grade fitness trackers given to participant to record their steps are shown in Table 1. Smartphone-paired trackers acted as interface to receive immediate and continuous information on individual PA. Real-time collection and uploading of step count data was done by participants syncing their fitness trackers using the H365 app and on-going analysis was conducted by HPB.

### Measures and instruments

The main outcome of interest was the daily step count recorded by participants’ fitness trackers and consistency in their daily wear. The devices used have shown to provide reliable and objective step counts (Table 1) [46]*.* The intervention’s reach was defined by the number of eligible participants who registered for the intervention and paired their devices. Requisite governance and regulatory approvals were obtained for the collection and confidential use of routine data. Step data was collected in accordance with Singapore’s Personal Data Protection Act (PDPA) and anonymized for the data analyses.

Pre-intervention participant characteristics for each wave were collected at registration. Self-reported variables included age, gender and baseline minutes of PA/week and Asian body mass index (aBMI) [12]. Post-intervention surveys were conducted by phone among randomly selected respondents at the end of the Challenge (*n*=2000) and 4 months after the Challenge (*n=*200) to assess step maintenance.

### Statistical analysis

Data were uploaded daily, cleaned, screened for invalid entries and analysed by a dedicated team at HPB. Analyses were conducted for participants completing the Challenge (guaranteed rewards period) with a 50,000 daily step upper bound in the tracker and H365 app to cap daily step counts and manage outliers. Very low step counts were excluded as a result of the positive correlation between daily step counts and completing the Challenge. Means and standard deviations were calculated for continuous variables and proportions and 95% confidence intervals (95%CI) for dichotomous variables. Baseline characteristics in the intervention population were compared to the resident population of Singapore using a two-tailed z-score significance test. Mean daily step counts at baseline (pre-intervention launch) and during the Challenge were compared using a t-test of significance in Stata v.13 and effect sizes calculated [47]. We used t-tests rather than repeated measures ANOVA for several reasons, including the observational nature of our data with uneven responses, comparisons limited to wave 3 and 1, and the large and diverse population sample enrolled. Given the observational nature of the data, responses measured at different points in each participant’s journey resulted in unbalanced numbers of repeats across individuals, which may lead to data being dropped in the analysis; similarly missing data on the response variable could result in entire cases being dropped. Sub-group of new and loyal participants (who registered for three waves) were also examined. The Standards for Reporting on Implementation Studies (StaRI) were adhered to and a checklist is attached in Supplementary material Table 1 [48].

## Results

### Population recruitment and characteristics

A total of 1,184,410 *unique* individuals aged 17 years or older enrolled for at least one of three waves of the Challenge (Table 2) out of 1,210,733 sign-ups (Table 3). The largest proportion were aged between 17 and 39 years, decreasing slightly in wave 3 (w1: 53%, w2: 48% and w3: 46%, respectively). One fifth was between 40 and 49 years old with the smallest group being 60 years or older (w1: 9.5%; w2: 16% and w3: 18%). Over half the participants were female, a trend consistent across the three waves (w1:59%, w2:58% and w3:58%). Nearly half of respondents reported moderate-to high-body mass indices (≥23 kg/m^2^) (w1: 53%, w2: 48% and w3: 48%, respectively), however the non-response on this question was high in wave 1. The proportion of participants reporting < 150 min of weekly PA at baseline increased from 15% in wave 1 to 21% in wave 3 but remained lower than the overall Singapore population (26%). Baseline characteristics are based on responses from 78% of registered participants in wave 1, 85% in wave 2 and 89% in wave 3 (Table 2).

**Table 2:** Characteristics of NSC 1 to NSC 3 participants and the general Singapore population

Baseline characteristics	Wave 1 (7/11/15–31/7/2016)		Wave 2 (1/10/16–31/3/2017)		Wave 3 (28/10/17–30/4/2018)		Singapore 2017	
	n	%	n	%	n	%	%	(95% CI)
Total individuals signed-up	129,677	83%	364,500	100%	690,233	100%	n/a	n/a
Participants surveyed at baseline	101,000	78%	311,000	85%	611,000	89%	n/a	n/a
**Gender**								
Female	59,590	59%*	180,380	58%*	354,380	58%*	51%	(50.0–52.0)
Male	41,410	41%	130,620	42%	256,620	42%	49%	(48.0–50.0)
**Age**								
17–39 years	53,530	53%*	149,280	48%*	281,060	46%*	39%	(38.0–40.0)
40–49 years	19,190	19%	62,200	20%	128,310	21%*	19%	(18.2–19.8)
50–59 years	16,160	16%*	49,760	16%*	103,870	17%*	19%	(18.2–19.8)
60–69 years	9090	9%*	34,210	11%*	73,320	12%*	15%	(14.3–15.7)
≥ 70 years	3030	0.5%*	15,550	5%*	36,660	6%	7%	(6.5–7.5)
Body Mass Index**(kg/m^*2*^*)*								
High risk (≥27.5)	11,898	19%	39,310	16%*	81,141	16%*	18%	(17.2–18.8)
Moderate risk (23–27.4)	21,291	34%	78,621	32%*	162,282	32%*	35%	(34.0–36.0)
Low risk (18.5–22.9)	25,048	40%	98,276	40%	192,709	38%*	40%	(39.0–41.0)
Low weight (≤ 18.5)	3757	6%*	29,483	12%*	70,998	14%*	8%	(7.4–8.6)
Non-response on bmi question	38,380	38%	65,310	21%	103,870	17%	n/a	n/a
**Baseline physical activity**								
< 150 min/week (inactive)	15,150	15%*	14,140	14%*	91,650	21%*	26%	(25.1–26.9)
**Daily step count** *(number, sd)*	4512	(4135)	6221	(5400)	7432	(5012)		
% < 5000 steps/day (sedentary)	533	21%	326	14%	310	15%	n/a	n/a
% ≥ 5000 steps/day (active)	1969	79%	2017	86%	1716	85%	n/a	n/a

*Source:* Health Promotion Board, 2019. Department of Statistics, 2018. *NSC* National Step Challenge, *n/a* not applicable, *CI* Confidence Interval, *sd* standard deviation. *indicates a *P*-value < 0.001 for a two-tailed significance test with 95% Confidence. ** Asian Body Mass Index (in kg/m^2^). Each wave begins on the first day of recruitment and tracks daily steps until the last day of the lucky draw

**Table 3:** Enrolment cascade and physical activity outcomes (NSC 1 to NSC 3)

	Wave 1		Wave 2		Wave 3		Total	
	n	% (sd)	n	% (sd)	n	% (sd)	n	%
**Enrolment cascade**								
Total number *sign-ups* ^(1)^	156,000	100%	364,500	100%	690,233	100%	**1,210,733**	**100%**
Total *unique* ^(1)^ individuals enrolled	129,677	83%	364,500	100%	690,233	100%	**1,184,410**	**98%**
Total new participants	129,677	83%	169,695	47%	487,369	71%	**927,947**	**78%**
Paired and used tracker	77,806	60%	233,280	64%	448,651	65%	**759,738**	**64%**
Paired but no tracker use or never paired	51,871	40%	131,220	36%	241,582	35%	**424,673**	**36%**
Challenge^a^ completers (end of rewards)	36,000	28%	150,000	88%	266,000	55%	**452,000**	**57%**
*Repeat* participants (any 2 waves)	32,645	25%	141,206	20%	41,306	3%	**215,157**	**23%**
*Loyal* participants (all 3 waves)	n/a		n/a		41,306		**41,306**	**4%**
**Physical Activity Outcomes**								
Tracker worn pre-Challenge (days/week)	2.4	(2.3)	3.9	(2.4)	5.3	(1.5)	**n/a**	**n/a**
Tracker worn in Challenge (days/week)	5.0	(2.2)	5.3	(2.1)	6.0	(2.0)	**n/a**	**n/a**
Daily steps during Challenge (mean, s d)	8675	(5505)	8463	(8463)	9077	(5270)	**n/a**	**n/a**
Mean difference daily steps (mean, sd)	4163	(1360)	2242	(334)	1645	(54)	**n/a**	**n/a**

*Source:* Health Promotion Board, 2019. *sd* standard deviation, *n/a *not applicable. ^a^ Challenge=guaranteed rewards period. The proportion of individuals who registered for the Step Challenge, who paired and wore the fitness tracker consistently during the period provided measures of *Reach*. *Effectiveness* was measured by the consistency in daily wear of the tracking device and the change in mean numbers of daily steps over time, illustrated in Figs. 1 and 2. *Maintenance* was measured by the extent to which physical activity and practice became established and routine among participants after the intervention. ^(1)^ In wave 1, 83% of all *sign-ups* were validated as *unique individuals* and duplicate entries were excluded. Improvements to the registration system in waves 2 and 3 prevented duplicate enrolment

Compared to the Singapore population, significantly more females, 18–39 and 60–69 year-olds participated in the Challenge although differences narrowed between wave 1 and 3 (Table 2; Supplementary material Table 2)*.* The proportion of participants with moderate or high BMI was comparable to the general population in wave 1 and decreased in waves 2 and 3.

### Population uptake across waves

The reach of the intervention increased fourfold over the three waves from 129,677 participants in wave 1 (2015–16) to 690,233 in wave 3 (2017–18). Two-thirds or more of registered participants effectively synced their fitness trackers with H365 in each wave (w1: 60%, w2: 64% and w3: 65%). The Challenge was completed by 28% of participants in wave 1, 88% in wave 2 and 55% in wave 3. Recruitment and follow-up are shown in Table 3. Among those completing the Challenge, daily step counts above the threshold accounted for 0.3% of all data points and for 3% of these participants, with at least one data point count above 50,000. Participants who registered for two waves or more accounted for 23% of all registrations (20% in wave 2, 25% in wave 3) while a small cohort of 41,306 people participated in all 3 waves.

**Fig. 1:**
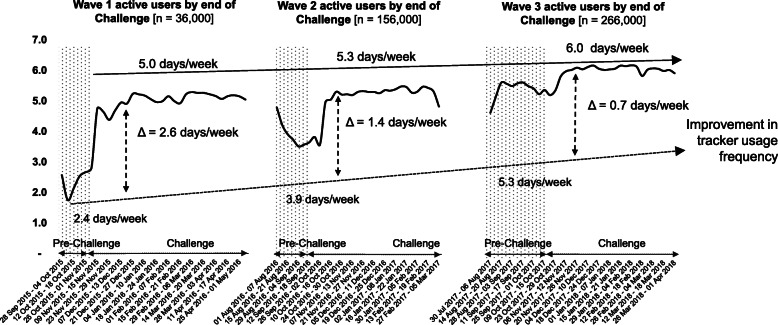
Weekly fitness tracker use between pre-Challenge and Challenge periods across waves. NSC=National Step Challenge. Pre-Challenge=period between registration and the start of the challenges and rewards. Challenge period=period during which step count-based rewards can be obtained (guaranteed or sure-win and lucky-draw). Δ=difference between pre-Challenge and Challenge days of tracker use per week

The frequency of fitness tracker use increased by 2.6 days/week among participants completing the Challenge in wave 1 (from 2.4 to 5.0 days/week) (Table 3; Fig. 1). In wave 2, tracker use between pre-Challenge and Challenge periods increased by 1.4 days/week (from 3.9 to 5.3 days/week) and in wave 3 by 0.7 days/week (from 5.3 to 6.0 days/week) (Table 3; Fig. 1). The proportion of participants using their fitness tracker throughout the Challenge period increased by 6% between waves 1 and 3 (54 to 62%).

**Fig. 2:**
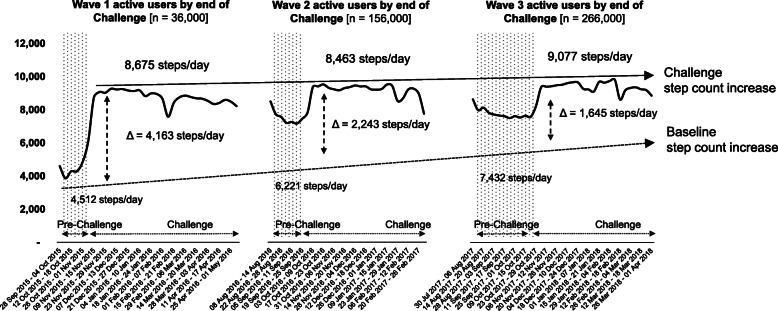
Increase in daily steps between pre-Challenge and Challenge periods across waves. NSC=National Step Challenge. Pre-Challenge=period between registration and the start of the Challenge. Challenge period=period during which step count-based rewards can be obtained (guaranteed or sure-win and lucky-draw). Δ=difference between pre-Challenge and Challenge mean daily steps

Mean daily steps among Challenge completers increased by 4163 (sd=1360; *p*< 0.001) in wave 1 (from 4512 to 8675 steps/day), by 2242 (sd=334; p< 0.001) in wave 2 (6221 to 8463 steps/day) and by 1645 (sd=54; *p*< 0.001) in wave 3 (7432 to 9077 steps/day) (Table 3; Fig. 2; Supplementary Material Table 3). The mean differences in daily steps between pre-Challenge and Challenge period narrowed markedly between waves, suggesting improvements in both baseline and Challenge period steps. Between waves 1 and 3, mean pre-Challenge daily steps increased by 2920 (from 4512 to 7432; *p*< 0.001) and by 402 during the Challenge (8675 to 9077; p< 0.001).

Sub-group analyses among “new” and “loyal” participants (who joined all three waves) displayed similar trends in step increases within and across waves (Table 4). Mean daily steps among new participants increased by 4163 (sd=1360; p< 0.001) between pre-Challenge and Challenge period in wave 1, by 2242 (sd=334; p< 0.001) in wave 2 and by 1645 (sd=54; p< 0.001) in wave 3. Mean daily steps among “loyal” participants increased by 5427 (sd=451; p< 0.001) in wave 1, by 1888 (sd=1072; p< 0.001) in wave 2 and by 1587 (sd=479; p< 0.001) in wave 3 (Table 4; Supplementary material Table 3).

**Table 4:** Physical Activity Outcomes by sub-group and wave (NSC 1 to NSC 3)

	Wave 1				Wave 2				Wave 3			
	n/mean	sd	t-stat	***P-***value	n/mean	sd	t-stat	***P-***value	n/mean	sd	t-stat	***P-***value
Total individuals signed-up	129,677	n/a	n/a	n/a	364,500	n/a	n/a	n/a	690,233	n/a	n/a	n/a
Total new participants	129,677	n/a	n/a	n/a	169,695	n/a	n/a	n/a	487,369	n/a	n/a	n/a
**All participants**^a^	36,000				150,000				266,000			
Daily baseline steps (all participants)	4512	(4135)	*143.5*	p< 0.001	6221	(5400)	*171.4*	p< 0.001	7432	(5012)	*161.0*	p< 0.001
Daily steps completers *(all participants)*	8675	(5505)			8463	(5066)			9077	(5066)		
Step increase completers (Δ) *(all participants)*	4163	(1360)			2242	(334)			1645	(54)		
**New participants**	36,000				115,000				114,000			
Daily baseline steps *(new participants)*	4512	(4135)	*143.5*	p< 0.001	6207	(5400)	*150.7*	*p*< 0.001	7132	(3706)	*120.4*	p< 0.001
Daily steps completers *(new participants)*	8675	(5505)			8429	(5066)			8915	(5066)		
Step increase completers (Δ) *(new participants)*	4163	(1360)			2222	(334)			1783	(1360)		
**Loyal**^b^ **participants**					38,000				38,000			
Daily baseline steps *(loyal participants)*	4545	(3769)	*205.9*	p< 0.001	8115	(3839)	*73.6*	p< 0.001	8424	(3695)	*61.9*	p< 0.001
Daily steps completers *(loyal participants)*	9972	(4220)			10,003	(2767)			10,011	(3216)		
Step increase completers (Δ) *(loyal participants)*	5427	(451)			1888	(1072)			1587	(479)		

*Source:* Health Promotion Board, 2019. *n* sample size, *sd* standard deviation, *CI* Confidence Interval, *n/a* Not Applicable

^a ^who synced until end of the Challenge period. “All” participants include new and loyal participants

^b ^“Loyal” participants took part in all three waves

Post-intervention maintenance was assessed for each wave, and across successive waves. The survey conducted in a sub-sample showed that 83% of physically inactive participants at baseline had become active during the Step Challenge and 4 months after the Challenge ended, 67% sustained increased PA [49]. The majority (93%) of participants who were already physically active at baseline remained active 4 months after the Challenge had ended.

## Discussion

This public health experiment demonstrated that translating proven interventions from controlled study settings to a real-world environment, combining them in a mutually reinforcing way and scaling up to achieve population-level change is feasible and effective, under certain conditions.

Over a million resident adults of all ages engaged with this intervention, a sizeable proportion of whom increased their PA and remained engaged. Marked increases in daily fitness tracker use between registration and Challenge and over three waves also suggested increased awareness, interest and adoption of tracking steps and PA.

Evidence of habituation was reflected in consistent increases in daily steps, within waves and across waves, in all sub-groups. Daily steps increased steadily between waves 1 and 3 while the difference between participants’ pre- and during-Challenge daily steps dropped. Increases within waves and across all three waves suggest that regular PA was maintained at population level and perhaps normalised among those habituated to tracking daily steps and incidental walking. These findings align with those of population surveys where awake-time spent by Singaporean adults on incidental PA increased from 5% in 2010 to 14% in 2017 and from 5 to 10% on moderate-intensity activity in the same period [50].

Four intervention cornerstones, integrated within the physical environment, contributed to high population-level participation. The first was the use of technology which strengthened data analytics and contributed to increase the immediacy of nudges and positive reinforcement for incremental activity. Second, the fun element introduced by behavioural economics and gamification increased appeal whereas the wrist-worn devices provided visible prompts and publicity. Third, the marketing campaign announcing the Challenge and device activation events spiked interest and created visibility, reinforced by queues lining up for free wearable trackers. Evidence reviews have shown that linking media campaigns to community programmes is effective and this may have contributed to the large numbers of people engaging in this experiment [17]. Finally, connecting the intervention to communities in an existing health-promoting environment facilitated the uptake and maintenance of incidental walking. Improved walkability was an important intervention enabler and consistent with findings that well-designed urban environments effectively contributed to increased PA [51–53]. Over 120,000 participants registered for the first Challenge, more than double the 50,000 expected and planned for. Adoption of this population-based intervention was facilitated by its geographical reach, alternative option of pairing one’s own device using H365 and prompt customer support for troubleshooting. These measures made it available to all residents, communities and localities without restrictions and the unexpected surge in registrations attest to its attractiveness.

Integrating intervention components and harnessing the potential of timely data analytics were central to maximising its reach, effectiveness, adoption and maintenance. Customised cues and rewards generated from individual data kept participants engaged, while continuous monitoring and analysis of aggregate cohort data were used to adjust implementation modalities throughout the intervention. Among others, they informed reward distribution patterns, amount and intervals, and the introduction of monthly thematic challenges [21, 49]. First wave analytics for example, showed that participants rapidly increased their daily steps to obtain rewards until the end of the guaranteed period, then slowed down once incentives were no longer provided. Based on observations, rewards were spread over a longer period of several months during which habituation was expected to occur [35, 43]; monthly thematic challenges were introduced to maintain interest and motivation at times when these seemed to wane. For example, data analytics suggested that, contrary to expectations, people remained physically active during festive periods such as the Chinese New Year but slacked off after festivities, prompting the introduction of a thematic challenge after major festive periods. Scale and strong data analytics also provided opportunities to test new modalities, including moderate to vigorous physical activity (MVPA) in a sub-group of participants*.*

Increased daily steps within and across waves is consistent with incidental PA and suggests habituation. Elsewhere, incidental PA was found to provide an entry point and manageable first step to increase population-level PA [17, 54]. Incidental PA is especially relevant for people citing lack of time as the main reason for not engaging in leisure-time PA [10]. Studies have shown the health benefits of even modest increases in daily steps, consistently associated with decreased mortality, especially among older men and women [40, 55–58]*.*

Noteworthy implementation observations included sharing of Challenge activities by participants on social media, which may have contributed to reinforcing its social dimensions and indirectly, motivation [43]. Studies elsewhere found social connections and enabling physical environments to be important factors facilitating continuation as well as uptake of PA [17, 52, 59, 60]*.*

Most studies of lifestyle behaviour show that the strongest effect occurs in the period shortly after intervention with a follow-up duration frequently insufficient to determine long-term behavioural trajectories [54]. Having successfully engaged a million adults to increase PA, maintaining the gains achieved will require sustained efforts. Strategies to support long-term behavioural change include strengthening links between the Step Challenge and other physical and online platforms for PA, including referrals to leisure time/fitness physical activities, while systematically integrating PA considerations in Singapore’s urban planning [54, 61, 62]. Further detailed analyses also aim to identify sub-group patterns, unmet needs or gaps to increase the intervention’s reach and impact.

The mixed results reported by some multi-component interventions attest to the challenges of resourcing, implementing and evaluating complex public health interventions on this scale, and to the subtle balance between implementation science and delivery. This experiment designed to increase population-level PA, was comprehensive and instructive despite not having benefitted from controlled study conditions, since intervention allocation could not be withheld to or from particular communities or areas [63]*.* While the absence of comparison group in this real-world intervention is a limitation, sub-group analyses were conducted to explore possible differences in outcomes. Our approach was strengthened by integrating rigorous and timely data analytics and methods throughout the intervention.

Participant baseline characteristics differed from Singapore’s adult population and cannot be assumed to represent the total population. In addition, characteristics at enrolment such as weight and height (used to derive aBMI) were self-reported and may have been subject to social desirability bias and/or under-reporting as seen in the high non-response rate in wave 1. Given the possible bias towards under reporting of weight, baseline BMI may have been underestimated and while this may not affect study outcomes, findings may apply to a heavier population. The self-selection of participants may also introduce bias where an intervention attracts a group of interested participants who may be more active and responsive (eg. “the worried well”). The trade-off between rapidly scaling up population-wide physical activity and collecting detailed data from individual participants, led to data on potential confounders not being collected and adjusted for. It is possible that education, socio-economic status, social support or exogenous factors such as a nationwide diabetes prevention campaign may also have influenced the uptake of PA. We acknowledge these limitations. However, the sheer scale of engagement with the Challenge over time suggested that it was effective in increasing PA at the population-level. In-depth analyses are planned to identify and characterise population sub-groups with low uptake and/or engagement to understand and address specific challenges, barriers and/or unmet needs and help tailor future intervention waves.

## Conclusions

Reversing unhealthy behaviours linked to diabetes, cancers and cardio-vascular disease is a public health priority which a population-level experiment like this can offer learnings on, for Asia and beyond. Originally designed to increase PA on a large scale, the intervention successfully mobilised a cohort of participants and harnessed technology and data analytics for public health. Future work will also measure the intensity of PA to differentiate moderate-to-vigorous from gentle PA and on maximising the engagement of individuals and groups at risk but not engaging with, interventions.

## Supplementary Information

**Additional file 1: Table S1.** Standards for Reporting Implementation Studies: the StaRI checklist. **Table S2.** Baseline characteristics: NSC participants and the general population (NSC 1 to NSC 3). **Table S3.** Physical activity outcomes by sub-group and wave.

## Data Availability

The datasets generated and analysed during the current study are not publicly available due to them containing information that could compromise participant privacy/consent. However, they may be made available from the authors on an anonymised basis and on reasonable request.

## Abbreviations

aBMI: Asian Body Mass Index
BMI: Body Mass Index
CI: Confidence Intervals
HPB: Health Promotion Board
NCD: Non-communicable Diseases
NSC: National Step Challenge
PA: Physical activity
MVPA: Moderate to Vigorous Physical Activity
PDPA: Personal Data Protection Act

## Footnotes

1 Based on WHO recommended minimum 150 min weekly physical activity. A 2010 survey found that 10% of students engaged in less than 30 min of any physical activity per week (NPHS, 2010).

2 A 2014 study estimated that nine out of ten Singaporean residents aged 16–45 used a smartphone (Deloitte TMT, 2015).

## References

[1] Kohl HW, Craig CL, Lambert EV, Inoue S, Alkandari JR, Leetongin G (2012). The pandemic of physical inactivity: global action for public health. Lancet.

[2] Ekelund U, Steene-Johannessen J, Brown WJ, Fagerland MW, Owen N, Powell KE (2016). Does physical activity attenuate, or even eliminate, the detrimental association of sitting time with mortality? A harmonised meta-analysis of data from more than 1 million men and women. Lancet.

[3] Andersen LB, Mota J, Di Pietro L (2016). Update on the global pandemic of physical inactivity. Lancet..

[4] Global Burden of Disease Study DALY and HALE Collaborators (2017). Global, regional, and national disability-adjusted life-years (DALYs) for 333 diseases and injuries and healthy life expectancy (HALE) for 195 countries and territories, 1990–2016: a systematic analysis for the Global Burden of Disease Study 2016. Lancet (London, England).

[5] Roth GA, Abate D, Abate KH, Abay SM, Abbafati C, Abbasi N (2018). Global, regional, and national age-sex-specific mortality for 282 causes of death in 195 countries and territories, 1980–2017: a systematic analysis for the global burden of disease study 2017. Lancet.

[6] Ministry of Health (2019). Singapore, Institute for Health Metrics and Evaluation. The Burden of Disease in Singapore, 1990–2017: An overview of the global burden of disease study 2017 results.

[7] Ding D, Lawson KD, Kolbe-Alexander TL, Finkelstein EA, Katzmarzyk PT, van Mechelen W (2016). The economic burden of physical inactivity: a global analysis of major non-communicable diseases. Lancet.

[8] Bloom DE, Cafiero ET, Jané-Llopis E, Abrahams-Gessel S, Bloom LR, Fathima S (2011). The global economic burden of noncommunicable diseases.

[9] David E, Bloom EC, Jané-Llopis E, Abrahams-Gessel S, Bloom LR, Fathima S, et al. The Global Economic Burden of Noncommunicable Diseases, PGDA Working Papers 8712, Program on the Global Demography of Aging: Harvard School of Public Health; 2012.

[10] Ministry of Health S (2010). National Health Survey.

[11] Ministry of Health Singapore, Health Promotion Board Singapore (2014). The Healthy Living Master Plan.

[12] Organization WH (2004). Global strategy on diet, physical activity and health.

[13] Nocon M, Muller-Riemenschneider F, Nitzschke K, Willich SN (2010). Review article: increasing physical activity with point-of-choice prompts--a systematic review. Scand J Public Health.

[14] Baker PRA, Francis DP, Soares J, Weightman AL, Foster C. Community wide interventions for increasing physical activity. Cochrane Database Syst Rev. 2015;1. 10.1002/14651858.CD008366.pub3 PMC9508615 25556970

[15] Kahn EB, Ramsey LT, Brownson RC, Heath GW, Howze EH, Powell KE (2002). The effectiveness of interventions to increase physical activity. A systematic review. Am J Prev Med.

[16] Organization WH (2010). Global recommendations on physical activity for health.

[17] Heath GW, Parra DC, Sarmiento OL, Andersen LB, Owen N, Goenka S (2012). Evidence-based intervention in physical activity: lessons from around the world. Lancet..

[18] Mitchell MS, Goodman JM, Alter DA, John LK, Oh PI, Pakosh MT (2013). Financial incentives for exercise adherence in adults: systematic review and meta-analysis. Am J Prev Med.

[19] Finkelstein EA, Haaland BA, Bilger M, Sahasranaman A, Sloan RA, Nang EEK (2016). Effectiveness of activity trackers with and without incentives to increase physical activity (TRIPPA): a randomised controlled trial. Lancet Diabetes Endocrinol.

[20] Kramer JN, Tinschert P, Scholz U, Fleisch E, Kowatsch T (2019). A cluster-randomized trial on small incentives to promote physical activity. Am J Prev Med.

[21] Monroe CM (2016). Valuable steps ahead: promoting physical activity with wearables and incentives. Lancet Diabetes Endocrinol.

[22] Howe KB, Suharlim C, Ueda P, Howe D, Kawachi I, Rimm EB (2016). Gotta catch'em all! Pokemon GO and physical activity among young adults: difference in differences study. BMJ.

[23] Bravata D, Smith-Spangler C, Sundaram V, Gienger A L, Lin N, Lewis R (2007). Using Pedometers to Increase Physical Activity and Improve Health: A Systematic Review.

[24] Deloitte Global Technology MaTT (2017). Mobile multiplies: global Mobile consumer survey, southeast Asia edition.

[25] Afshin A, Babalola D, Mclean M, Yu Z, Ma W, Chen CY, Arabi M, Mozaffarian D. Information technology and lifestyle: a systematic evaluation of internet and mobile interventions for improving diet, physical activity, obesity, tobacco, and alcohol use. J Am Heart Assoc. 2016;5(9):e003058. 10.1161/JAHA.115.003058 PMC5079005 27581172

[26] Board HP (2019). Health promotion Board Mission Singapore: health promotion Board.

[27] Birken SA, Powell BJ, Shea CM, Haines ER, Alexis Kirk M, Leeman J (2017). Criteria for selecting implementation science theories and frameworks: results from an international survey. Implement Sci.

[28] Theobald S, Brandes N, Gyapong M, El-Saharty S, Proctor E, Diaz T (2018). Implementation research: new imperatives and opportunities in global health. Lancet..

[29] Peters DH, Adam T, Alonge O, Agyepong IA, Tran N (2013). Implementation research: what it is and how to do it. BMJ.

[30] Dahlgren G, Whitehead M (1991). Policies and strategies to promote social equity in health.

[31] Stokols D (1996). Translating social ecological theory into guidelines for community health promotion. Am J Health Promot.

[32] McLaren L, Hawe P (2005). Ecological perspectives in health research. J Epidemiol Community Health.

[33] Sallis JF, Cervero RB, Ascher W, Henderson KA, Kraft MK, Kerr J (2006). An ecological approach to creating active living communities. Annu Rev Public Health.

[34] Sallis JF, Linton L, Kraft MK (2005). The first active living research conference: growth of a transdisciplinary field. Am J Prev Med.

[35] Duhigg C (2012). The power of habit: why we do what we do in life and business: Random House.

[36] Charpentier CJ, Bromberg-Martin ES, Sharot T (2018). Valuation of knowledge and ignorance in mesolimbic reward circuitry. Proc Natl Acad Sci.

[37] Owain Service (2012). Michael Hallsworth DHFA, Rory Gallagher, Sam Nguyen, Simon Ruda, Michael Sanders, with Marcos Pelenur AG, Hugo Harper, Joanne Reinhard & Elspeth Kirkman.

[38] Chatterton T, Wilson C (2014). The ‘four dimensions of behaviour’ framework: a tool for characterising behaviours to help design better interventions. Transp Plan Technol.

[39] Tudor-Locke C, Craig CL, Brown WJ, Clemes SA, De Cocker K, Giles-Corti B (2011). How many steps/day are enough? For adults. Int J Behav Nutr Phys Act.

[40] Lee IM, Shiroma EJ, Kamada M, Bassett DR, Matthews CE, Buring JE. Association of step volume and intensity with all-cause mortality in older women. JAMA Intern Med. 2019;179(8):1105–12. 10.1001/jamainternmed.2019.0899 PMC6547157 31141585

[41] Sanders M, Snijders V, Hallsworth M (2018). Behavioural science and policy: where are we now and where are we going?. Behav Public Policy.

[42] Howe KB, Suharlim C, Ueda P, Howe D, Kawachi I, Rimm EB (2016). Gotta catch’em all! Pokémon GO and physical activity among young adults: difference in differences study. BMJ.

[43] Board HP (2019). Internal programme documents.

[44] The Straits Times Rachel Au-Yong (2018). Walk2Ride scheme to extend walkways will hit 200km milestone on Sept 19. The Straits Times.

[45] Board NP (2017). Nature nurtures: Annual Report 2017/2018.

[46] Evenson KR, Goto MM, Furberg RD (2015). Systematic review of the validity and reliability of consumer-wearable activity trackers. Int J Behav Nutr Phys Act.

[47] StataCorp (2015). Stata Statistical Software: Release 13.

[48] Pinnock H, Barwick M, Carpenter CR, Eldridge S, Grandes G, Griffiths CJ (2017). Standards for reporting implementation studies (StaRI) statement. BMJ..

[49] Health Promotion Board S (2019). HPB monitoring and evaluation data.

[50] Ministry of Health HPB, Singapore (2019). National Population Health Survey.

[51] National Institute for Clinical Excellence (2018). Physical activity and the environment.

[52] National Institute for Clinical Excellence. Promoting and creating built or natural environments that encourage and support physical activity (NICE public health guidance 8). United Kingdom. 2008.

[53] Sallis JF, Cerin E, Conway TL, Adams MA, Frank LD, Pratt M (2016). Physical activity in relation to urban environments in 14 cities worldwide: a cross-sectional study. Lancet.

[54] Ory MG, Lee Smith M, Mier N, Wernicke MM (2010). The science of sustaining health behavior change: the health maintenance consortium. Am J Health Behav.

[55] Dwyer T, Pezic A, Sun C, Cochrane J, Venn A, Srikanth V (2015). Correction: objectively measured daily steps and subsequent long term all-cause mortality: the Tasped prospective cohort study. PLoS One.

[56] Dwyer T, Pezic A, Sun C, Cochrane J, Venn A, Srikanth V (2015). Objectively measured daily steps and subsequent long term all-cause mortality: the Tasped prospective cohort study. PLoS One.

[57] Jefferis BJ, Parsons TJ, Sartini C, Ash S, Lennon LT, Papacosta O (2018). Objectively measured physical activity, sedentary behaviour and all-cause mortality in older men: does volume of activity matter more than pattern of accumulation?. Br J Sports Med.

[58] Yamamoto N, Miyazaki H, Shimada M, Nakagawa N, Sawada SS, Nishimuta M (2018). Daily step count and all-cause mortality in a sample of Japanese elderly people: a cohort study. BMC Public Health.

[59] Heath GW, Brownson RC, Kruger J, Miles R, Powell KE, Ramsey LT (2006). The effectiveness of urban design and land use and transport policies and practices to increase physical activity: a systematic review. J Phys Act Health.

[60] National Institute for Clinical Excellence (2019). Physical activity: Overview of pathways.

[61] Urban Redevelopment Authority (2014). Singapore Urban Redevelopment Master Plan.

[62] Urban Redevelopment Authority (2019). Singapore Urban Redevelopment Master Plan 2019.

[63] Petticrew M, Cummins S, Ferrell C, Findlay A, Higgins C, Hoy C (2005). Natural experiments: an underused tool for public health?. Public Health.

