# Supplementary material for: Can a multi-level intervention approach, combining behavioural disciplines, novel technology and incentives increase physical activity at population-level?
Source: BMC Public Health. 2021 Jan 11;21:120. doi: 10.1186/s12889-020-10092-x (PMC7802355; doi:10.1186/s12889-020-10092-x)
Supplement: Supplementary file 1 — Additional file 1: Table S1. Standards for Reporting Implementation Studies: the StaRI checklist. Table S2. Baseline characteristics: NSC participants and the general population (NSC 1 to NSC 3). Table S3. Physical activity outcomes by sub-group and wave. [file 12889_2020_10092_MOESM1_ESM.docx]

**Can a novel approach using multi-level interventions, combining behavioural disciplines, technology and incentives increase physical activity at population-level?**

Authors: Ling CHEW^1 (†)^; Isabel TAVITIAN-EXLEY^1 (†)*^; Nicole LIM^1^; Alice ONG^1^

^1^ Health Promotion Board, Singapore

*Corresponding author

**SUPPLEMENTARY MATERIAL**

**Supplementary material. Table 1. Standards for Reporting Implementation Studies: the StaRI checklist**

| **Checklist item** | | **Reported on page #** | **Implementation Strategy** | **Reported on page #** | **Intervention** |
| --- | --- | --- | --- | --- | --- |
|  | |  | “Implementation strategy” = how intervention was implemented |  | “Intervention” = public health intervention being implemented. |
| **Title and abstract** | | | | | |
| Title | **1** | p1,2 | **Can a novel approach using multi-level interventions, combining behavioural disciplines, technology and incentives increase physical activity at population-level?** | | |
| Abstract | **2** | p1 | Identification as an implementation study, including a description of the implementation strategy to be tested, the evidence-based intervention being implemented, and defining the key implementation and health outcomes. | | |
| **Introduction** | | | | | |
| Introduction | **3** | P3 | Description of the problem, challenge or deficiency in healthcare or public health that the intervention being implemented aims to address. | | |
| Rationale | **4** | P3 | Scientific background and rationale for the implementation strategy (including any underpinning theory/ framework/ model, how it is expected to achieve effects and pilot work). | p3,4 | The scientific background and rationale for the intervention being implemented (including evidence about its effectiveness and how it is expected to achieve its effects). |
| Aims & objectives | **5** | P4 | The aims of the study, differentiating between implementation objectives and any intervention objectives. | | |
| **Methods: description** | | | | | |
| Design | **6** | P4 | The design and key features of the evaluation, (cross referencing to any appropriate methodology reporting standards) and any changes to study protocol, with reasons | | |
| Context | **7** | p3,4,5 | The context in which the intervention was implemented. (Consider social, economic, policy, healthcare, organisational barriers and facilitators that might influence implementation elsewhere). | | |
| Targeted ‘sites’ | **8** | P4,5 | The characteristics of the targeted ‘site(s)’ (e.g locations/ personnel/ resources etc.) for implementation and any eligibility criteria. | P4 | The population targeted by the intervention and any eligibility criteria. |
| Description | **9** | p4,5 | A description of the implementation strategy | p5,6 | A description of the intervention |
| Sub-groups | **10** | P6 | Any sub-groups recruited for additional research tasks, and/or nested studies are described | | |
| **Methods: evaluation** | | | | | |
| Outcomes | **11** | p5,6 | Defined pre-specified primary and other outcome(s) of the implementation strategy, and how they were assessed. Document any pre-determined targets | p5,6 | Defined pre-specified primary and other outcome(s) of the intervention (if assessed), and how they were assessed. Document any pre-determined targets |
| Process evaluation | **12** | P4,5 | Process evaluation objectives and outcomes related to the mechanism by which the strategy is expected to work | | |
| Economic evaluation | **13** | Not covered in this paper | Methods for resource use, costs, economic outcomes and analysis for the implementation strategy | Not covered in this paper | Methods for resource use, costs, economic outcomes and analysis for the intervention |
| Sample size | **14** | p4 | Rationale for sample sizes (including sample size calculations, budgetary constraints, practical considerations, data saturation, as appropriate) | | |
| Analysis | **15** | p5,6 | Methods of analysis (with reasons for that choice) | | |
| Sub-group analyses | **16** | P6 | Any a priori sub-group analyses (e.g. between different sites in a multicentre study, different clinical or demographic populations), and sub-groups recruited to specific nested research tasks | | |
| **Results** | | | | | |
| Characteristics | **17** | p6,7 and SuppMat | Proportion recruited and characteristics of the recipient population for the implementation strategy | P6,7 and  SuppMat | Proportion recruited and characteristics (if appropriate) of the recipient population for the intervention |
| Outcomes | **18** | P6,7 | Primary and other outcome(s) of the implementation strategy | P6,7 | Primary and other outcome(s) of the Intervention (if assessed) |
| Process outcomes | **19** | P7,8 | Process data related to the implementation strategy mapped to the mechanism by which the strategy is expected to work | | |
| Economic evaluation | **20** | Not covered in this paper | Resource use, costs, economic outcomes and analysis for the implementation strategy | Not covered in this paper | Resource use, costs, economic outcomes and analysis for the intervention |
| Sub-group analyses | **21** | p6,7 | Representativeness and outcomes of subgroups including those recruited to specific research tasks | | |
| Fidelity/ adaptation | **22** | p7,8 | Fidelity to implementation strategy as planned and adaptation to suit context and preferences | p7,8 | Fidelity to delivering the core components of intervention (where measured) |
| Contextual changes | **23** | P8 | Contextual changes (if any) which may have affected outcomes | | |
| Harms | **24** | P8 | All important harms or unintended effects in each group | | |
| **Discussion** | | | | | |
| Structured discussion | **25** | p8,9,10 | Summary of findings, strengths and limitations, comparisons with other studies, conclusions and implications | | |
| Implications | **26** | p9,10 | Discussion of policy, practice and/or research implications of the implementation strategy (specifically including scalability) | p9,10 | Discussion of policy, practice and/or research implications of the intervention (specifically including sustainability) |
| **General** | | | | | |
| Statements | **27** | P6 | Include statement(s) on regulatory approvals (including, as appropriate, ethical approval, confidential use of routine data, governance approval), trial/study registration (availability of protocol), funding and conflicts of interest | | |

Note: Requisite governance and regulatory approvals were obtained for the collection and confidential use of routine data. Step data was collected in accordance with Singapore’s personal data protection act (PDPA), anonymised and unlinked for the data analyses.

**Supplementary material Table 2. Baseline characteristics of participants and the general population** (NSC 1 to NSC 3)

|  | **Wave 1** | | | |  | **Wave 2** | | | |  | **Wave 3** | | | |  | **Singapore** | | |  |  |  |  |  |  |
| --- | --- | --- | --- | --- | --- | --- | --- | --- | --- | --- | --- | --- | --- | --- | --- | --- | --- | --- | --- | --- | --- | --- | --- | --- |
|  | 7/11/15-31/7/2016 | | 95% CI | |  | 1/10/16-31/3/2017 | | 95% CI | |  | 28/10/17-30/4/2018 | | 95% CI | |  |  | 95% CI | | **W1** | | **W2** | | **W3** | |
|  | n | % | ll | ul |  | n | % | ll | ul |  | n | % | ll | ul |  | % | ll | ul | z-test | P-value | z-test | P-value | z-test | P-value |
| Total individuals signed-up | 129,677 | 83% | (82.9% | 83.3%) |  | 364,500 | 100% | - | - |  | 690,233 | 100% | - | - |  | **n/a** |  |  | *-* |  | *-* |  | *-* |  |
| Participants surveyed | 101,000 | 78% | (77.6% | 78.1%) |  | 311,000 | 85% | (85.2% | 85.1%) |  | 611,000 | 89% | (88.4% | 88.6%) |  | **n/a** |  |  | *-* |  | *-* |  | *-* |  |
| **Gender** |  |  |  |  |  |  |  |  |  |  |  |  |  |  |  |  |  |  |  |  |  |  |  |  |
| Female | 59,590 | 59% | (58.7% | 59.3%) | ***** | 180,380 | 58% | (57.8% | 58.2%) | * | 354,380 | 58% | (57.9% | 58.1%) | * | **51%** | (50.0% | 52.0%) | *14.8* | <0.0001 | *13.3* | <0.0001 | *13.4* | <0.0001 |
| Male | 41,410 | 41% | (40.7% | 41.3%) |  | 130,620 | 42% | (41.8% | 42.2%) |  | 256,620 | 42% | (41.9% | 42.1%) |  | **49%** | (48.0% | 50.0%) | *-* |  | *-* |  | *-* |  |
| **Age** |  |  |  |  |  |  |  |  |  |  |  |  |  |  |  |  |  |  |  |  |  |  |  |  |
| 18-39 years | 53,530 | 53% | (52.7% | 53.3%) | ***** | 149,280 | 48% | (47.8% | 48.2%) | * | 281,060 | 46% | (45.9% | 46.1%) | * | **39%** | (38.0% | 40.0%) | *1000* | <0.0001 | *7000* | <0.0001 | *224.4* | <0.0001 |
| 40-49 years | 19,190 | 19% | (18.8% | 19.2%) |  | 62,200 | 20% | (19.9% | 20.1%) |  | 128,310 | 21% | (20.9% | 21.1%) | * | **19%** | (18.2% | 19.8%) | *-* |  | *-* |  | *-* |  |
| 50-59 years | 16,160 | 16% | (15.8% | 16.2%) |  | 49,760 | 16% | (15.9% | 16.1%) | * | 103,870 | 17% | (16.9% | 17.1%) | * | **19%** | (18.2% | 19.8%) | *-* |  | *-* |  | *-* |  |
| 60-69 years | 9,090 | 9.0% | (8.8% | 9.2%) | ***** | 34,210 | 11% | (10.6% | 11.4%) | * | 73,320 | 12% | (11.9% | 12.1%) | * | **15%** | (14.3% | 15.7%) | *-* |  | *-* |  | *-* |  |
| ≥ 70 years | 3,030 | 0.5% | (0.5% | 0.5%) | ***** | 15,550 | 5% | (4.9% | 5.1%) | * | 36,660 | 6% | (5.9% | 6.1%) |  | **7%** | (6.5% | 7.5%) | *-* |  | *-* |  | *-* |  |
| **Body Mass Index**** (kg/m3) | |  |  |  |  |  |  |  |  |  |  |  |  |  |  |  |  |  |  |  |  |  |  |  |
| High risk (≥27.5) | 11,898 | 19% | (18.8% | 19.2%) |  | 39,310 | 16% | (15.9% | 16.1%) | * | 81,141 | 16% | (15.9% | 16.1%) | * | **18%** | (17.2% | 18.8%) | *317.6* | <0.0001 | *172.6* | <0.0001 | *20.3* | <0.0001 |
| Moderate risk (23-27.4) | 21,291 | 34% | (33.4% | 34.6%) |  | 78,621 | 32% | (31.8% | 32.2%) | * | 162,282 | 32% | (31.9% | 32.1%) | * | **35%** | (34.0% | 36.0%) | *-* |  | *-* |  | *-* |  |
| Low risk (18.5-22.9) | 25,048 | 40% | (39.7% | 40.3%) |  | 98,276 | 40% | (39.8% | 40.2%) |  | 192,709 | 38% | (37.9% | 38.1%) | * | **40%** | (39.0% | 41.0%) | *-* |  | *-* |  | *-* |  |
| Low weight (≤ 18.5) | 3,757 | 6% | (5.9% | 6.1%) | ***** | 29,483 | 12% | (11.9% | 12.1%) | * | 70,998 | 14% | (13.9% | 14.1%) | * | **8%** | (7.4% | 8.6%) | *-* |  | *-* |  | *-* |  |
| *Non-response on question* | *38,380* | *38%* | *(37.7%* | *38.3%)* |  | *65,310* | *21%* | (20.9% | 21.1%) |  | *103,870* | *17%* | *(16.9%* | *17.1%)* |  |  |  |  | *-* |  | *-* |  | *-* |  |
| **Baseline physical activity** |  |  |  |  |  |  |  |  |  |  |  |  |  |  |  |  |  |  |  |  |  |  |  |  |
| < 150 minutes/week | 15,150 | 15% | (14.8% | 15.2%) | ***** | 43,540 | 14% | (13.9% | 14.1%) | * | 91,650 | 21% | (20.9% | 21.1%) | * | **26%** | (25.1% | 26.9%) | *27.3* | <0.0001 | *32.0* | <0.0001 | *11.5* | <0.0001 |
| ≥ 150 minutes/week | 85,850 | 85% | (84.8% | 85.2%) |  | 267,460 | 86% | (85.9% | 86.1%) |  | 519,350 | 79% | (78.9% | 79.1%) |  | **74%** | (73.1% | 74.9%) | *-* |  | *-* |  | *-* |  |
| Daily steps (mean number, *sd*) | 4,512 | *4,135* |  |  |  | 6,221 | *5,400* |  |  |  | 7,432 | *5,012* |  |  |  | *n/a* |  |  | *-* |  | *-* |  | *-* |  |

*Source:* Health Promotion Board, 2019. Department of Statistics, 2018. NSC=National Step Challenge. n/a=not applicable. CI= Confidence Interval. ll=lower limit; ul=upper limit. *indicates a P-value <0.001 for a two-tailed significance test with 95% confidence. ** Asian Body Mass Index. Each wave begins on the first day of recruitment and ends tracking steps on the last day of the lucky draw.

**Supplementary material Table 3. Physical activity outcomes by sub-group and wave**

|  | **Wave 1** | | | | | | | **Wave 2** | | | | | | **Wave 3** | | | | | | **Wave 3_1** | |
| --- | --- | --- | --- | --- | --- | --- | --- | --- | --- | --- | --- | --- | --- | --- | --- | --- | --- | --- | --- | --- | --- |
|  | **n/ mean** | **(sd)** | **95%CI** | |  | **t-stat** | **P-value** | **n/mean** | **(sd)** | **95%CI** | | **t-stat** | **P-value** | **n/mean** | **(sd)** | **95%CI** | | **t-stat** | **P-value** | **t-stat** | **P-value** |
| Total *unique* participants signed-up | 129,677 | - | - | - |  | na | na | 364,500 | - | - |  | na | na | 690,233 | - | - | - | na | na | na | na |
| Total new, unique participants | 129,677 | - | - | - |  | na | na | 169,695 | - | - |  | na | na | 487,369 | - | - | - | na | na | na | na |
| **All participants** *completers****** | 36,000 |  |  |  |  |  |  | 150,000 |  |  |  |  |  | 266,000 |  |  |  |  |  |  |  |
| Daily baseline steps (all participants) | 4,512 | (4,135) | 4,384 | 4,640 |  | *143.5* | p<0.001 | 6,221 | (5,400) | 6,194 | 6,248 | *171.4* | p<0.001 | 7,432 | (5,012) | 7,413 | 7,451 | *161.0* | p<0.001 | *300.5* | *p<0.001* |
| Daily steps completers *(all participants)* | 8,675 | (5,505) | 4,469 | 4,555 |  |  |  | 8,463 | (5,066) | 8,437 | 8,489 |  |  | 9,077 | (5,066) | 9,057 | 9,097 |  |  | *60.1* | *p<0.001* |
| Step increase completers (Δ)*(all participants)* | 4,163 | (1,360) | 8,618 | 8,732 |  |  |  | 2,242 | (334) | 2,240 | 2,244 |  |  | 1,645 | (54) | 1,645 | 1,645 |  |  |  |  |
| **New participants** | 36,000 |  |  |  |  |  |  | 115,000 |  |  |  |  |  | 114,000 |  |  |  |  |  |  |  |
| Daily baseline steps *(new participants)* | 4,512 | (4,135) | 4,469 | 4,555 |  | *143.5* | p<0.001 | 6,207 | (5,400) | 6,176 | 6,238 | *150.7* | p<0.001 | 7,132 | (3,706) | 7,110 | 7,154 | *120.4* | p<0.001 | *196.7* | *p<0.001* |
| Daily steps completers *(new participants)* | 8,675 | (5,505) | 8,618 | 8,732 |  |  |  | 8,429 | (5,066) | 8,400 | 8,458 |  |  | 8,915 | (5,066) | 8,886 | 8,944 |  |  | *15.4* | *p<0.001* |
| Step increase completers (Δ)*(new participants)* | 4,163 | (1,360) | 4,149 | 4,177 |  |  |  | 2,222 | (334) | 2,220 | 2,224 |  |  | 1,783 | (1,360) | 1,775 | 1,791 |  |  |  |  |
| **Loyal** participants** *completers* | - | - |  |  |  |  |  | 38,000 |  |  |  |  |  | 38,000 |  |  |  |  |  |  |  |
| Daily baseline steps *(loyal participants)* | 4,545 | (3,769) | 4,506 | 4,584 |  | *205.9* | p<0.001 | 8,115 | (3,839) | 8,076 | 8,154 | *73.6* | p<0.001 | 8,424 | (3,695) | 8,387 | 8,461 | *61.9* | p<0.001 | 143.5 | *p<0.001* |
| Daily steps completers *(loyal participants)* | 9,972 | (4,220) | 9,928 | 10,016 |  |  |  | 10,003 | (2,767) | 9,975 | 10,031 |  |  | 10,011 | (3,216) | 9,979 | 10,043 |  |  | 1.4 | *p=0.075* |
| Step increase completers (Δ)*(loyal participants)* | 5,427 | (451) | 5,422 | 5,432 |  |  |  | 1,888 | (1,072) | 1,877 | 1,899 |  |  | 1,587 | (479) | 1,582 | 1,592 |  |  |  |  |

*Source:* Health Promotion Board, 2019. * Completers were participants who synced until end of guaranteed rewards period. “All” participants include new and loyal participants. **“Loyal” participants took part in all three waves. n=sample size. sd=standard deviation. CI=Confidence Interval.
